# Description and molecular characterization of *Geraldius jejuensis* n. sp. (Nematoda: Chambersiellidae) from Korea

**DOI:** 10.2478/jofnem-2025-0023

**Published:** 2025-06-21

**Authors:** Abraham Okki Mwamula, Chang-hwan Bae, Dae Geun Lee, Yi Seul Kim, Yeong-Don Lee, Dong Woon Lee

**Affiliations:** Research Institute of Invertebrate Vector, Kyungpook National University, Sangju 37224, Republic of Korea; Biodiversity Research Department, Species Diversity Research Division, National Institute of Biological Resources, Incheon, 22689, Republic of Korea; Department of Ecological Science, Kyungpook National University, Sangju, 37224, Republic of Korea; Hallasan Research Department, World Heritage Office, Jeju Special Self-Governing Province, Jeju. 63143, Republic of Korea

**Keywords:** *Diastolaimus*, *Geraldius*, molecular characterization, morphology, morphometrics, new species, phylogeny, taxonomy

## Abstract

A new species of the genus Geraldius isolated from the wood of a dead black pine tree is characterized using morphological data and molecular DNA barcodes. *Geraldius jejuensis* n. sp. is characterized by its lateral fields with two incisures; lip region conoid to rounded and continuous with body; hemizonid and excretory pore located posterior to nerve ring; excretory pore opening just at the beginning of hemizonid or within the contour of hemizonid; vulva a transverse slit in ventral view; opening in a depression, creating a circular profile in lateral view; rectum 1.4 to 1.7 times longer than anal body diameter; phasmids located 55.0 to 78.5 μm from anal opening; tail elongated, 146.0 to 177.0 μm long; gubernaculum 27.0 to 33.5 μm long, caudal papillae arrangement of seven pairs pre-cloacal, two adcloacal, and six post-cloacal; and three additional midventral papillae on anterior cloacal lip. The new species was compared with the three known species of the genus, including *G. bakeri, G. galapagoensis* and *G. inserrai*. The phylogenetic relationships among species were reconstructed using 18S-rRNA and 28S-rRNA gene sequences. Inferences from both genes corroborate the close morphological relationships between *Geraldius* and *Diastolaimus*.

The genus *Geraldius* was proposed by Sanwal (1971) to describe a single species whose morphological characters deviated from the type of *Chambersiella* Cobb, 1920. *Geraldius* Sanwal, 1971 distinguished species characterized by cephalic cirri and two ovaries from the closely related genera *Chambersiella* and *Diastolaimus* Rahm, 1928. Until recently, the genus has been monotypic, with only one known species — *Geraldius bakeri* (Sanwal, 1957) Sanwal, 1971. *Geraldius bakeri* was recovered and described from the bank of the Jock River, near Richmond, in Ontario, Canada. Outside of its type locality, it has only been reported in Costa Rica from lichens hanging from a tree (Holovachov et al., 2003). In 2012, Cid del Prado (2012) isolated the second species of the genus *G. galapagoensis* Cid del Prado, 2012 from mosses of the family Meteoriaceae growing in endemic trees *Scalesia pedunculata* Hook in the tropical forest of Galápagos Islands, Ecuador. Cid del Prado-Vera et al., (2021) isolated the third species — *Geraldius inserrai* Cid Del Prado-Vera, Ferris and Subbotin, 2021 — from lichens and epiphytic plants growing on branches of trees in Mexico State, México. Thus, species of this genus have only been reported in the Americas (North America and South America).

During a nematological survey conducted in 2025 in pine forest ecosystems in the Republic of Korea, a population of an undescribed species belonging to *Geraldius* was recovered from the bark layer of a dead pinewood in a nematode-infected black pine tree stand (*Pinus thunbergii* Parl). The new species, herein designated as *Geraldius jejuensis* n. sp., is described, considering both morphological and molecular DNA phylogenetic comparisons.

## Materials and Methods

### Nematode population and extraction

The population was recovered from the bark layer of a dead pinewood nematode-infected black pine, *Pinus thunbergii* sampled from Jeju Island, Jeju province, Republic of Korea. Nematodes were extracted from bark layer cuttings using the Baermann funnel method (Baermann, 1917). The nematode suspension was examined under a Nikon SMZ 1000 stereomicroscope (Nikon), and nematode specimens belonging to the genus *Geraldius* were hand-picked and characterized according to diagnostic criteria based on morphometric data and molecular DNA barcodes.

### Morphological characterization

Seventeen female and 19 male individuals were heat-killed, fixed in formalin-glycerin proportion, and processed to pure glycerin according to Seinhorst (1959), as modified by De Grisse (1969). Photomicrographs and morphometric data were taken using a Zeiss imager Z2 microscope (Carl Zeiss) fitted with Axio-vision, a material science software for research and engineering (Carl Zeiss). Line drawings were made under a drawing tube and redrawn using CorelDRAW software version 24 (Alludo). Species description was done following the diagnostic species descriptions presented by Sanwal (1957), (1971), and Cid del Prado (2012), and morphometric data were compared with the only three known species of the genus.

### Molecular characterization

DNA was extracted from heat-relaxed, morphometrically-confirmed single female and male specimens following the protocol described by Iwahori et al. (2000). Polymerase chain reaction (PCR) was performed using WizPure Taq DNA Polymerase kit (Wizbiosolutions, Gyeonggi-do, Korea) in accordance with the manufacturer's instructions. Two gene fragments — 18S-rRNA gene and the D2-D3 expansion segment of 28S-rRNA gene — were amplified and sequenced. The 18S-rRNA gene was amplified as two partially overlapping fragments using two sets of primers: 988F (5′-CTCAAAGATTAAGCCATGC-3′) and 1912R (5′-TTTACGGTCAGAACTAGGG-3′), and 1813F (5′-CTGCGTGAGAGGTGAAAT-3′) and 2646R (5′-GCTACCTTGTTACGACTTTT-3′) (Holterman et al., 2006). The D2-D3 expansion segment of the 28S-rRNA gene was amplified using the primer sets D2A (5′-ACAAGTACCGTGAGGGAAAGTTG-3′) and D3B (5′-TCGGAAGGAACCAGCTACTA-3′) (Nunn, 1992). Polymerase chain reaction was executed with a Bio-Rad T100 PCR cycler (Bio-Rad, Hercules, CA), and the thermal profiles for the above primer sets were assessed as described by Mwamula et al. (2023). The PCR products were subsequently purified using a QIAquick PCR Purification Kit (Qiagen Inc., Valencia, CA) and quantified using a quickdrop spectrophotometer (Molecular Devices, San Jose, CA). The purified products were directly sequenced in both directions with the same primers as specified above. The DNA sequencing process was conducted at Macrogen Inc. (Seoul, Korea). The obtained sequences were edited and submitted to the NCBI GenBank database under the following accession numbers: PV399962, PV399963 (for 18S-rRNA), and PV399959-PV399961 (for 28S-rRNA).

### Phylogenetic analysis

By using the BLAST homology search program, the obtained sequences (18S-rRNA and 28S-rRNA gene) were compared with those of related species, including comparable sequences of taxa from other related genera published in GenBank (Holovachov et al., 2013; Čermák et al., 2022; Cid del Prado-Vera et al., 2021, 2023). Multiple alignments for the two genes were assembled using ClustalX (Thompson et al., 1997). The sequences of *Alloionema appendiculatum* Schneider, 1859 (FJ665982), *Neoalloionema tricaudatum* Ivanova, Pham & Spiridonov, 2016 (KR817916), *Strongyloides robustus* Chandler, 1942 (AB272232) and *Rhabditophanes* sp. (AF202151) were used as the outgroup taxa for the 18S-rRNA gene. *Steinernema glaseri* (Steiner, 1929) Wouts, Mráček, Gerdin & Bedding, 1982 (KU180692) and *Necator americanus* Stiles, 1902 (KU1806941) were selected as the outgroup taxa for 28S-rRNA gene. Bayesian inference (BI) of the phylogenies was performed using MrBayes 3.2.7 (Ronquist et al., 2012) with the GTR + I + G model selected for the two datasets. Bayesian analysis was run with four chains for 1 × 10^6^ generations, with the Markov chains sampled at intervals of 100 generations. Consensus trees were produced with the 50% majority rule. The generated trees were edited using FigTree v1.4.4 software (Andrew Rambaut). Posterior probabilities (PP) exceeding 50% were given on applicable clades. Intraspecific and interspecific sequence variation were examined using PAUP* v4.0a169 (Swofford, 2003).

## Results

### Systematics

**Figure 1:**
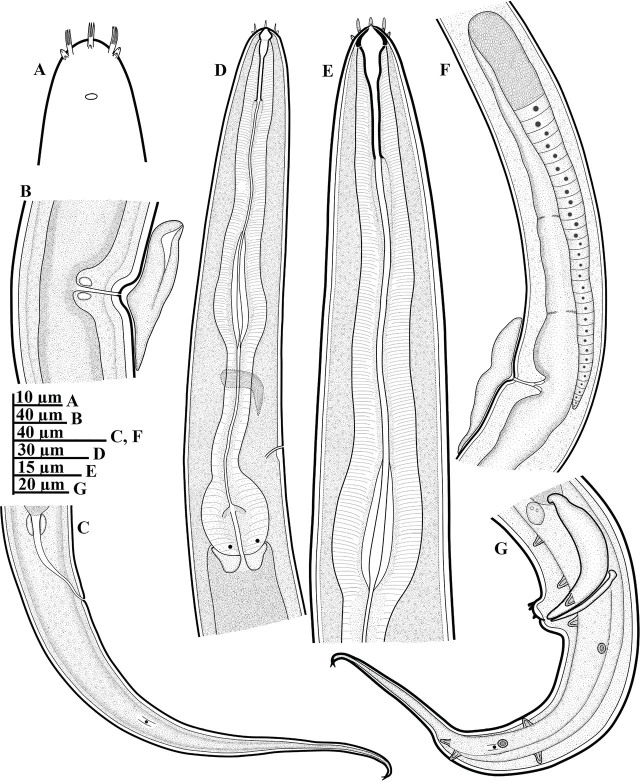
Line drawings of *Geraldius jejuensis* n. sp. (A–G): A: Anterior end, surface view; B: Vulval region; C: Female tail region; D: Anterior region with the entire esophagus; E: Anterior region with the corpus region; F: Female reproductive system; G: Male tail region, including the copulatory apparatus and arrangement of caudal papillae.

**Figure 2:**
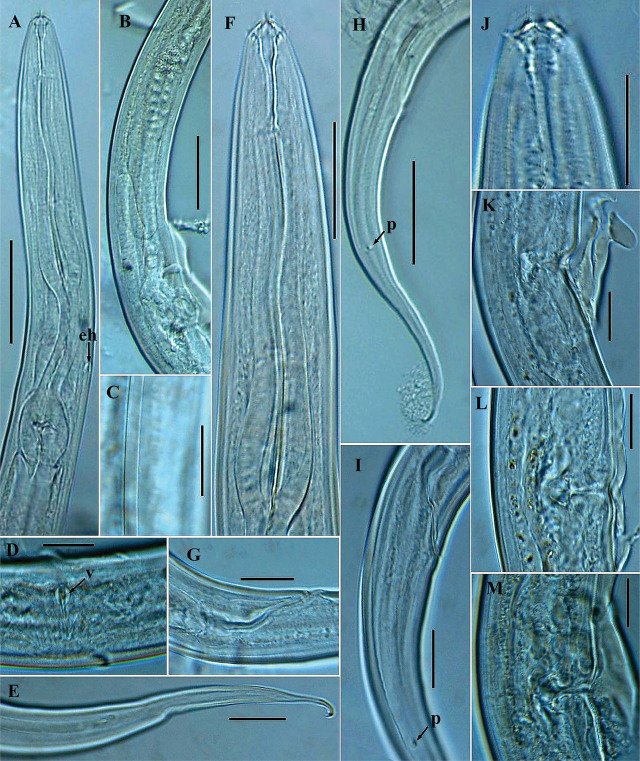
Photomicrographs of *Geraldius jejuensis* n. sp. (A–M). A, F: Female anterior region; B: Female reproductive system; C: Lateral field; D: Ventral view of the vulva; E: Posterior region of female tail; G: Rectum and anal region; H: Female tail region; I: Anterior region of female tail, including position of phasmids; J: Anterior region with cirri; K, L, M: Variation in vulval region in lateral view. The arrow labeled eh indicates the position of excretory pore and hemizonid; v = transverse vulva; p = phasmids (Scale bars: A, B, H = 50 μm; C, D, E, G, I, J, K, L and M = 20 μm; F = 30 μm).

**Figure 3:**
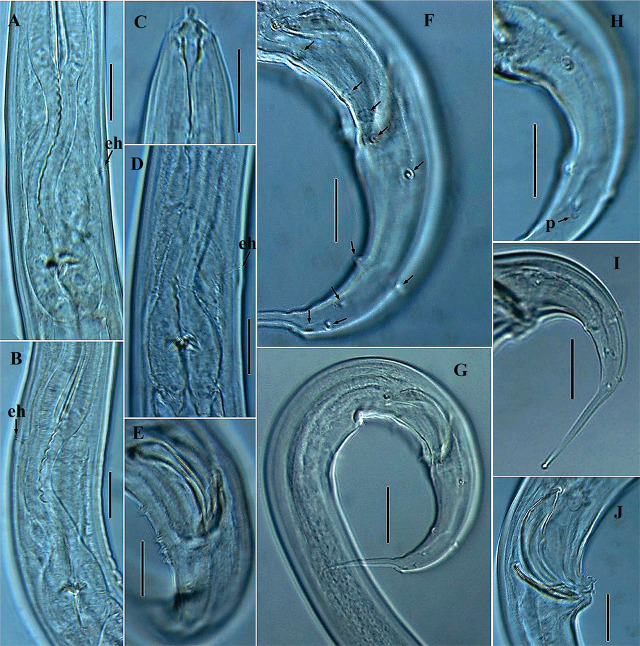
Photomicrographs of *Geraldius jejuensis* n. sp. (A–J). A, B, D: Posterior part of esophagus with variation in position of excretory pore and hemizonid; C: Male anterior region; E: Dorsal lateral view of the three papillae located in close proximity to cloaca; F: Arrangement of post–cloacal papillae, including the preceding trio; G, I: Male tail region with post–cloacal papillae arrangement; H: Arrangement of post–cloacal papillae in relation to phasmids; J: Male copulatory apparatus and cloacal opening with the three additional midventral papillae on anterior cloacal lip. The arrow labeled eh indicates the position of excretory pore and hemizonid; unlabeled arrows = position of papillae; p = phasmids (Scale bars: A, B, C, D, E, F, H, and J = 20 μm; G, I = 30 μm).

*Geraldius jejuensis* n. sp. (Table 1; Figs. 1, 2 & 3).

**Table 1:** Morphometrics of *Geraldius jejuensis* n. sp. from Korea.

**Character**	**Holotype** ♀	♀♀	♂♂
n		16	19
L	1187.0	1163.4±61.4 (1040.0–1306.0)	1112.8±92.7 (928.0–1255.0)
a	24.8	26.9±1.9 (23.6–30.3)	30.7±2.8 (26.8–36.5)
b	5.4	5.4±0.2 (4.9–5.9)	5.2±0.5 (4.1–6.0)
c	7.4	7.4±0.3 (7.0–7.9)	9.4±0.9 (8.1–11.0)
c'	6.2	6.3±0.4 (5.7–7.2)	3.7±0.4 (3.1–4.8)
V or T	48.2	48.0±0.7 (46.8–49.3)	64.2±3.9 (54.5–69.5)
G_1_	37.8	32.1±2.6 (27.6–37.8)	–
G_2_	30.9	30.0±3.9 (23.9–40.3)	–
Lip height	3.5	3.4±0.3 (3.0–4.0)	3.4±0.3 (3.0–4.0)
Lip diam.	10.5	10.5±0.5 (9.5–11.5)	10.3±0.6 (8.5–11.0)
Cheilostom length	5.5	5.0±0.6 (4.0–6.0)	4.7±0.5 (4.0–5.5)
Cheilostom diam.	4.5	5.1±0.9 (4.0–6.5)	5.5±0.5 (4.5–6.5)
Gymnostom length	6.5	5.7±0.8 (4.5–7.5)	5.5±0.5 (4.5–7.0)
Stegostom length	18.5	18.2±1.4 (16.0–20.0)	18.2±0.9 (17.0–20.0)
Total stoma length	31.0	28.8±1.1 (27.0–31.0)	28.5±0.8 (27.0–30.0)
Amphidial opening	12.0	10.9±0.9 (9.5–13.5)	10.9±1.1 (9.0–13.5)
Corpus length	137.0	131.4±4.5 (123.5–140.0)	127.8±6.4 (112.0–142.5)
Metacorpal diam	21.5	20.4±0.8 (19.0–22.0)	19.3±1.1 (17.0–21.0)
Isthmus length	53.5	53.2±2.2 (50.5–58.0)	53.8±3.3 (48.0–59.0)
Corpus/isthmus	2.6	2.5±0.1 (2.4–2.7)	2.4±0.1 (2.2–2.6)
Anterior to nerve ring	146.0	145.0±6.7 (131.0–155.5)	135.6±6.4 (124.0–149.0)
Anterior to Ex. pore	156.0	164.9±9.1 (148.0–183.0)	151.6±11.5 (131.0–178.0)
Anterior to Deirid	162.0	165.4±8.2 (149.0–183.0)	151.4±11.5 (132.0–179.0)
Anterior to Hemizonid	156.0	163.9±8.5 (147.5–180.0)	151.1±11.4 (131.0–177.0)
Basal bulb length	31.5	33.8±1.3 (31.5–36.0)	33.5±1.9 (30.0–36.5)
Basal bulb diam.	25.0	24.6±0.7 (23.0–26.0)	23.7±1.7 (20.0–26.0)
Esophagus length	220.5	217.2±5.4 (207.0–227.0)	213.6±8.9 (193.0–229.5)
Cardia length	10.5	9.9±1.0 (7.0–11.5)	10.2±1.4 (7.5–12.0)
Maximum body diam.	48.0	43.3±3.2 (38.0–48.0)	36.4±3.5 (30.0–43.0)
Vulval body diam.	45.5	40.4±3.3 (33.5–45.5)	–
Rectum	36.0	38.5±2.7 (34.0–43.5)	–
Anal / cloacal body diam.	26.0	24.8±1.0 (22.5–27.0)	32.5±3.1 (28.0–39.0)
Phasmids to anus	59.0	64.8±6.4 (55.0–78.5)	55.0±4.6 (44.5–66.0)
Tail length	160.0	156.4±9.8 (146.0–177.0)	118.9±10.6 (101.0–135.0)
Phasmids/tail (%)	39.3	41.4±2.7 (36.7–46.6)	46.5±4.8 (40.2–54.2)
Spicules	–	–	54.3±2.6 (50.5–59.5)
Gubernaculum	–	–	30.7±1.7 (27.0–33.5)

All measurements are in μm and in the form of average ± SD (range). G_1_ and G_2_ were calculated using overall length of genital branches, including flexures.

### Description

*Female:* Body habitus generally C-shaped when heat-killed and fixed, gradually narrowing anteriorly from the neck region, and posteriorly from vulva region. Cuticle with very fine striae, annuli less than a μm apart. Lateral fields with two incisures, starting in the posterior part of corpus and ending posterior to phasmids. Lip region 9.5 to 11.5 μm in diameter, conoid to rounded anteriorly, continuous with body contour. Labial region with six lips, with six branched cirri ca. 4.0 to 5.5 μm long. Anterior sensilla are arranged in two circles: six outer setae and four cephalic setae. Amphidial aperture is located at level of the base of gymnostom, 9.5 to 13.5 μm from anterior end. Stoma 2.5 to 3.0 times as long as labial region diameter, sclerotized, clearly subdivided into three regions: cheilostom, gymnostom and stegostom. Cheilostom 4.0 to 6.0 μm long and 4.0 to 6.5 μm wide; gymnostom 4.5 to 7.5 μm long, narrower than cheilostom; stegostom 16.0 to 20.0 μm long, narrowing from anterior portion, continuing as an undivided tubular portion enveloped with conspicuous cuticular lining. Dorsal esophageal gland orifice located in the posterior third of the stegostom. Esophageal corpus is cylindrical anteriorly, with prominent sclerotized lining, and terminates into an enlarged metacorpal region with a prominent wide lumen but without a distinct median bulb. Esophageal isthmus is narrow, with thinner cuticular lining than the corpus. Basal bulb is oval, 31.5 to 36.0 μm long and 23.0 to 26.0 μm wide, with strongly developed valvular apparatus. Nuclei of esophageal glands are located in posterior region of basal bulb. Cardia is cylindroid, 7.0 to 11.5 μm long and envelops the preceding part of basal bulb. Nerve ring is variable, encircling esophagus in mostly the anterior part of isthmus, occasionally at mid-level of isthmus (the latter observed in four specimens) or, rarely, in posterior region (observed in two specimens). Hemizonid and excretory pore are visible, both located posterior to nerve ring and at posterior part of isthmus 147.5 to 183.0 μm from anterior end; rarely, located in the anterior portion of isthmus (the latter observed in two specimens, see Fig. 3B). Hemizonid is 4.0 to 6.0 μm long, with excretory pore opening just at the beginning of hemizonid or within the contour of hemizonid. Deirid is located inside lateral field, its position variable, at level of, and slightly anterior or posterior to, the excretory pore (between 5.0 μm anterior and 9.0 μm posterior to excretory pore level). Digestive system is simple. Reproductive system is didelphic and amphidelphic, with ovaries reflexed with oocytes arranged in a single profile. In some specimens, the reflexed portion of one of the ovaries spanning beyond the position of the vulva; others have the proximal part of especially the anterior ovary making a double flexure with the germinal zone being packed on right-hand side of the intestine. The oviduct is narrow in proximal part, and wide in the distal part. Uterus is simple, almost cylindroid, with no convolutions, possessing distinct walls and a visible lumen. Vagina occupies 38–48% of vulval body diameter. Small vulval glands are visible in some specimens. Vulva in mature females opens in a depression, giving a somewhat circular profile in lateral view (Fig. 1B and Figs. 2K, L). However, vulva is a transverse slit in ventral view (see Fig. 2D). Vulval opening and entire vulval region are often covered with a dense gelatin-like secretion, seemingly a copulatory plug. Rectum is prominent, 1.4 to 1.7 times longer than the anal body diameter, flanked by rectal glands. Anal opening is an arcuate slit. Phasmids are prominent, located anterior to middle of tail, 55.0 to 78.5 μm from anal opening, or at 37 to 47% of the tail length from the anus. Tail is elongated and conoid, narrowing gradually and ending in a sclerotized, dorsally curved and finely bifurcated hook-like tip.

*Male:* Generally are as abundant as females. General morphology is similar to that of female except for sexual characters and a more coiled tail region. Body habitus is generally C-shaped, with a ventrally coiled or strongly curved caudal region when heat-killed and fixed; body is generally slender than that of females. Lateral field appears narrower, with cephalic framework and lip region contour similar to that of females. Testis is single, outstretched in all examined specimens, bluntly ends at 33.0 to 60.0 μm from the basal bulb, and occupies 55 to 70% of the total body length. Spicules are paired and symmetrical, curved ventrally, each with a well-defined oval manubrium. Gubernaculum is prominent, 27.0 to 33.5 μm long, trough-like, and slightly ventrally curved at both ends, with a wide anterior part, reducing gradually to a narrow tip. Phasmids are located at 40 to 54% of the tail length from the cloaca.

Caudal papillae arranged as follows: seven pairs of latero-ventral pre-cloacal papillae, with the first pair located just short of cloacal opening, and close to the ensuing two adcloacal pairs, creating a trio appearance positioned equidistant to each other (see Fig. 3E). Post-cloacally, six pairs are present, arranged in the following positions: two lateral pairs (one pair just after cloacal position and another pair at the level of the phasmids); one ventrosublateral pair; and three dorsosublateral (with two of the three located almost at the end of the enlarged portion of the tail), as seen in arrangement in Fig. 1G and Fig. 3F. Additionally, three midventral papillae are present on anterior cloacal lip (two on the upper and one on the lower side, as seen in Fig. 3J). Tail is strongly curved ventrally and conoid, ending in a dorsally curved, sclerotized and finely bifurcated hook-like tip.

### Diagnosis and relationships

*Geraldius jejuensis* n. sp. is characterized by its lateral fields with two incisures; lip region conoid to rounded and continuous with body; hemizonid and excretory pore located posterior to nerve ring; excretory pore opening just at the beginning of hemizonid or within the contour of hemizonid; vulva a transverse slit in ventral view, opening in a depression, giving a circular profile in lateral view; rectum 1.4 to 1.7 times longer than anal body diameter; phasmids in female located at 55.0 to 78.5 μm from anal opening; tail elongated, 146.0 to 177.0 μm long; gubernaculum 27.0 to 33.5 μm long; caudal papillae arrangement: seven pairs pre-cloacal, two adcloacal, and six post-cloacal, with three additional midventral papillae on anterior cloacal lip.

*Geraldius jejuensis* n. sp. is closely similar to the three known species of the genus — *G. bakeri, G. galapagoensis* and *G. inserrai* — in the size of the body in females and males, and in the length and sclerotization of the stoma. It differs from *G. bakeri* by its more anterior vulval position (V = 46.8–49.3 vs. 48.8–53.0); vulva opening in a depression vs. vulva on elevated vulval cone; long tail (146.0–177.0 μm vs. 121.0–144.0 μm; c = 7.0–7.9 vs. 8.0–9.5); phasmids in females positioned more posteriorly from anal opening (55.0–78.5 μm vs. 33.0–46.0 μm); longer gubernaculum (27.0–33.5 μm vs. 24.0 μm); two pairs of adcloacal papillae located close to the first precloacal papillae; and 3 additional midventral papillae on anterior cloacal lip, as opposed to one pair of adcloacal and one additional midventral papilla on anterior cloacal lip.

It is distinguished from *G. galapagoensis* by its lip region continuous with body vs. marked by a constriction; longer esophagus (207.0–227.0 μm vs. 141.0–207.0 μm); longer isthmus (50.5–58.0 μm vs. 32.0–45.0 μm); longer distance of the excretory pore and deirid from the anterior end (148.0–183.0 μm vs. 110.0–145.0 μm; 149.0–183.0 μm vs. 129.0–152.0 μm, respectively); vulva opening in a depression vs. vulva on elevated vulval cone; long tail (146.0–177.0 μm vs. 81.0–121.0 μm and 101.0–135.0 μm vs. 78–98 μm in females and males, respectively); phasmids in females positioned more posteriorly from anal opening (55.0–78.5 μm vs. 32–51 μm), longer gubernaculum (27.0–33.5 μm vs. 20.0–25.0 μm); and in the number of post-cloacal caudal papillae: two adcloacal, six post-cloacal pairs, and three additional midventral papillae on anterior cloacal lip vs. three post-cloacal pairs, one pair lateroventral and two pairs dorsolateral.

It is distinguished from *G. inserrai* by its lip region continuous with body vs. marked by a constriction; vulva opening in a depression vs. vulva on elevated vulval cone; long tail (146.0–177.0 μm vs. 116.0–127.0 μm; 101.0–135.0 μm vs. 77.0–101.0 μm in females and males, respectively); phasmids in females positioned more posteriorly from anal opening (55.0–78.5 μm vs. 40.0–55.0 μm); longer spicules (50.5–59.5 μm vs. 39.0–45.0 μm); and longer gubernaculum 27.0–33.5 μm vs. 18.0–21.0 μm).

### Type habitat and locality

The type population was recovered from the bark layer (cork cambium, secondary cortex and phloem layers) of a dead pinewood from a nematode-infected black pine stand, *Pinus thunbergii*, sampled from Jeju Island, Jeju province, Republic of Korea (GPS coordinates: 33°15′34″N, 126°13′52″E).

### Type material

Holotype female, nine female, and 10 male paratypes were deposited in the National Institute of Biological Resources of Korea; seven female and nine male paratypes were deposited in the Nematode Collection of Kyungpook National University (KNU), Republic of Korea.

### Etymology

*Geraldius jejuensis* n. sp. was isolated from a pine tree forest on Jeju Island, Jeju Province, Republic of Korea. The species epithet *jejuensis* is derived from the name of the island, i.e., Jeju.

### Molecular characterization and phylogeny

The nearly full-length 18S-rRNA gene amplicons yielded fragments of approximately 1,700 bp. No intraspecific sequence variation was recorded in the two newly-obtained 18S-rRNA gene sequences (PV399962, PV399963) of the new species *Geraldius jejuensis* n. sp. In the 18S-rRNA gene phylogeny, *Geraldius jejuensis* n. sp. sequences were grouped in an independent, moderately supported clade with sequences of *Diastolaimus grossus* (a member species of a closely-related genus). The two new 18S-rRNA gene sequences of *Geraldius jejuensis* n. sp. (PV399962, PV399963) differed from *Diastolaimus grossus* sequences (KC242218, KU180670, and OM691517) by 67 to 71 bp (4.5–4.7%).

**Figure 4:**
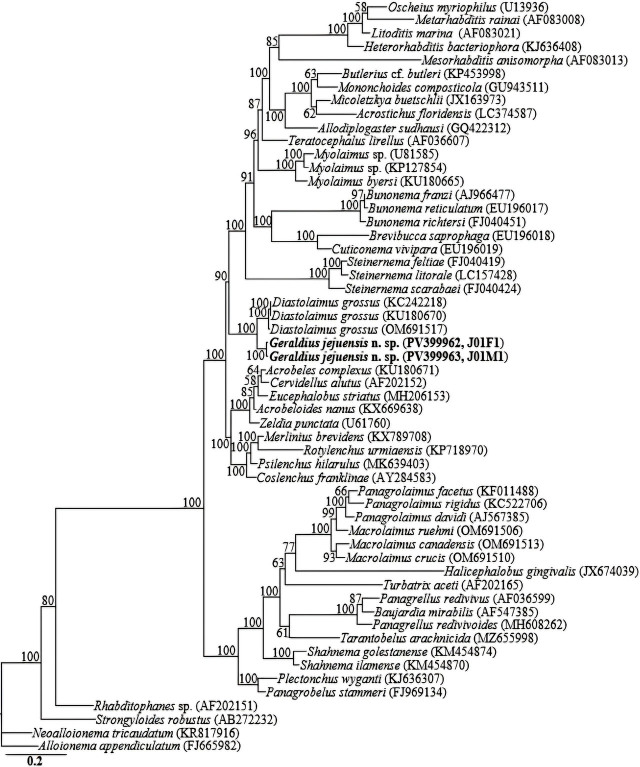
Bayesian tree inferred under the GTR + I + G model from 18S–rRNA gene sequences of *Geraldius* spp. and other species from various genera. Posterior probability values exceeding 50% are given on appropriate clades. The studied population is indicated in bold text. Outgroup taxa: *Alloionema appendiculatum*, *Neoalloionema tricaudatum*, *Strongyloides robustus* and *Rhabditophanes* sp.

**Figure 5:**
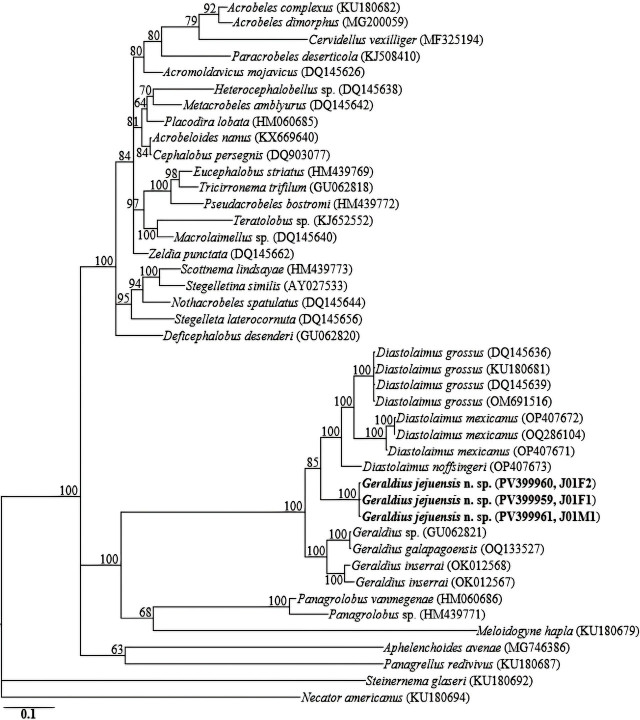
Bayesian tree inferred under the GTR + I + G model from LSU D2–D3 partial sequences of *Geraldius* spp. and other species from various genera. Posterior probability values exceeding 50% are given on appropriate clades. The studied population is indicated in bold text. Outgroup taxa: *Steinernema glaseri* and *Necator americanus.*

The D2-D3 region amplicons yielded fragments of approximately 750 bp. The three D2-D3 sequences of *Geraldius jejuensis* n. sp. (PV399959-PV399961) were also identical, with no intraspecific sequence variation. The generated sequences of *Geraldius jejuensis* n. sp. (PV399959-PV399961) showed relative homology with gene sequences of *Geraldius* spp. and *Diastolaimus* spp. in the GenBank database, all with percent identities of 85 to 88%, based on the BLAST homology search program. In the LSU phylogeny, the three *Geraldius jejuensis* n. sp. sequences were grouped in a well-supported (PP = 100%) independent clade with *Geraldius inserrai* (OK012567, OK012568), *Geraldius galapagoensis* (OQ133527), *Geraldius* sp. (GU062821), *Diastolaimus noffsingeri* (OP407673), *Diastolaimus grossus* (DQ145636, DQ145639, KU180681 and OM691516), and *Diastolaimus mexicanus* (OP407671, OP407672, and OQ286104), differing by 81 to 83 bp (11.8–12.6%), 85 bp (13.9%), 87 bp (11.8%), 76 bp (10.8%), 85 to 86 bp (11.5–12.4%) and 87 to 88 bp (12.3–12.7%), respectively. Fifty-six 18S-rRNA and 43 28S-rRNA gene sequences from member species of *Geraldius,* and other related nematode genera, including the newly obtained sequences and outgroup taxa, constituted the sequence dataset for phylogenetic analyses. Phylogenetic relationships as inferred from Bayesian analysis are shown in Figs. 4 and 5.

## Discussion

The two reconstructed phylogenies (18S-rRNA, and D2–D3 expansion of 28S-rRNA gene) indicate that *Geraldius jejuensis* n. sp. is genetically distinct from the available *Geraldius* gene sequences of two of the three species of the genus as represented by the Bayesian trees. Inferences from the more informative 28S-rRNA gene phylogeny corroborate the close morphological relationships between *Geraldius* and *Diastolaimus* detailed by Sanwal (1957, 1971). Morphologically, *Geraldius* can be differentiated from *Diastolaimus* by its well-developed cephalic cirri, and the posterior part of its stoma, which usually has conspicuously long, narrow-channeled stegostom, instead of a stegostom usually a shallow funnel-shaped lumen, enveloped by the anterior end of esophagus.

As noted by Yeates et al. (1993) and Cid Del Prado-Vera et al. (2021), nematodes of this group are classified as bacterivores because they have an open stoma without teeth. Members of the genus have predominantly been isolated from lichens and mosses of the family Meteoriaceae and epiphytic plants growing on branches of trees (Holovachov et al., 2003; Cid del Prado, 2012; Cid del Prado-Vera et al., 2021, 2023). However, some members of Chambersiellidae have been described as associated with insects, or with beetle galleries beneath tree barks (Massey, 1963, 1964). In our current findings, *Geraldius jejuensis* n. sp. was isolated from the outer layers (cork cambium, secondary cortex and phloem layers) of a pinewood nematode-infected dead pine tree. The wood had signs of insect infestation in the form of tunnel engravings of *Dendroctonus*, though no juvenile or adult forms of *Geraldius jejuensis* n. sp. were recovered from the *Dendroctonus* specimens collected from the wood tunnels. Nonetheless, such habitats are known to host and proliferate a variety of pinewood-colonizing bacteria and fungi, as well as bacteria that exploit insect cadavers (Nascimento et al., 2015; Alves et al., 2016). Therefore, as discussed by Cid del Prado-Vera et al. (2023), the relationship between nematodes of this group and insects may be phoretic and/or necromenic, with the nematodes exploiting the bacteria that proliferate in insect cadavers. Cid del Prado-Vera et al. (2021) observed both bacteria and fungal spores in the intestines of nematodes of the Chambersiellidae. Consequently, it would seem that members of the genus thrive on perhaps both bacteria and fungi. The genus has a restricted distribution (North America and South America) and none of the known species have been reported so far on other continents (Asia, Europe, Africa and Oceania). Therefore, this study extends the distribution of the genus to at least three continents.
